# Effects of actin remodeling inhibitors on cellular energy metabolism of a model marine bivalve, the Pacific oyster

**DOI:** 10.1242/jeb.249708

**Published:** 2025-04-25

**Authors:** Eugene P. Sokolov, Inna M. Sokolova

**Affiliations:** ^1^ Department of Marine Biology, Institute for Biological Sciences, University of Rostock, 18059 Rostock, Germany; ^2^ Department of Maritime Systems, Interdisciplinary Faculty, University of Rostock, 18059 Rostock, Germany

**Keywords:** Cytoskeleton, Actin treadmilling, Energy costs, Mitochondria, Proton leak, Bioenergetics, *Crassostrea gigas*, *Magallana gigas*

## Abstract

Actin, the most abundant cellular protein, is essential for maintaining structural organization, mechanical stability and cellular motility. The actin cytoskeleton undergoes continuous ATP-dependent reorganization, incurring significant energy costs through treadmilling. However, experimental quantifications of these energy expenditures, especially in ectotherms, remain scarce. In this study, we assessed the energy costs of actin remodeling in the Pacific oyster *Crassostrea* [also *Magallana*] *gigas*, a marine bivalve, by measuring oxygen consumption in the presence of inhibitors of actin treadmilling (latrunculin B, jasplakinolide and cytochalasin D). Our results indicate that under normal physiological conditions, actin remodeling contributes less than 5% to the cellular energy budget in gill and mantle cells of oysters. Unexpectedly, cytochalasin D induced a marked increase in mitochondrial proton leak, observed both in intact cells and isolated mitochondria, suggesting a connection between actin disorganization and increased mitochondrial maintenance costs. Notably, jasplakinolide and latrunculin B, which inhibit actin treadmilling through different mechanisms from those of cytochalasin D, had no effect on mitochondrial respiration. This suggests that different mechanisms of actin cytoskeleton disruption can lead to distinct cellular outcomes. Given the significant role of proton leak in cellular respiration, these findings suggest that actin dynamics may play a crucial role in regulating mitochondrial metabolism, with broad implications for cellular energy costs. Further studies are needed to elucidate the underlying mechanisms of actin–mitochondria interactions and their broader relevance to the regulation of cellular metabolism in ectothermic species.

## Teaser

**Summary:** Actin treadmilling constitutes a minor energy cost in differentiated gill and mantle tissues of adult oysters.

## INTRODUCTION

Metabolic rate is a fundamental physiological trait that has major implications for life history, ecology and evolution of the organisms (Burger et al., 2019; Burton et al., 2011; Kooijman et al., 2020; van der Meer, 2006). Basal metabolic rate (BMR) or standard metabolic rate (SMR) reflects the minimal energy required for the maintenance of homeostatic processes essential for survival and thus has a high allocation priority in the organismal energy balance (Norin and Metcalfe, 2019; Sokolova, 2021). Basal maintenance costs in animals are largely (>90%) determined by the cellular ATP consumers such as ion transport, protein turnover, synthesis of metabolic intermediates and maintenance of intracellular structures (Darveau et al., 2002; Hulbert and Else, 2000; Norin and Metcalfe, 2019; Sokolova, 2021; Suarez and Darveau, 2005). Therefore, assessment of the cellular energy budgets is essential for understanding of the processes that determine the cost of living. Cellular energy budgets have been extensively studied in endotherms (Else et al., 2004; Hulbert and Else, 2000, 2005; Hulbert et al., 2002; Rolfe and Brown, 1997). In endotherms, ion transport (in particular, Na^+^-K^+^ ATPase and Ca^2+^ ATPase), protein turnover, gluconeogenesis and proton leak collectively accounting for >70% of ATP consumption in most cell types (Rolfe and Brown, 1997). The cellular energy budgets of ectotherms, including invertebrates, received decidedly less attention and require further investigation to determine processes contributing to the energy costs of basal maintenance (Cherkasov et al., 2006; Hulbert et al., 2002; Pace and Manahan, 2007; Pan et al., 2015; Sokolova, 2021).

Cytoskeleton remodeling is considered an important cellular ATP sink in animals (Sokolova, 2021). Actin is the most abundant protein in animal cells and it plays key roles in structural organization, mechanical stability and movement of the cell (Rottner et al., 2017; Xu et al., 2017). The actin cytoskeleton undergoes constant dynamic reorganization (called ‘treadmilling’) that involves addition of G-actin monomers to the barbed (plus) end of F-actin polymers and their release at the pointed (minus) end (Engl et al., 2017). ATP-bound actin monomers are added to the barbed end of the filament, upon which ATP is hydrolyzed, inorganic phosphate is released, and ADP remains trapped in the actin subunit (Pollard et al., 2000). As the filament ages, a ubiquitous actin-binding protein profilin exchanges ADP to ATP leading to actin dissociation and its recycling into the monomer pool (Pollard et al., 2000). The ATP dependence of the dynamic stability of actin cytoskeleton implies that actin remodeling may incur substantial energy costs to the cell depending on the rate of actin treadmilling (Bernstein and Bamburg, 2003; DeWane et al., 2021; Xu and Bretscher, 2014). To date, experimental assessments of the energy costs of actin remodeling in animal cells are scarce and to the best of our knowledge, limited to endotherms. In mammalian and avian neurons, actin turnover has been estimated to contribute ∼25–50% of the cellular oxygen consumption (Bernstein and Bamburg, 2003; Engl et al., 2017). In human granulocytes, actin dynamics accounted for ∼20% of the baseline (resting) oxygen consumption and ∼73% of the oxygen consumption in the activated state (Roos et al., 1976). To the best of our knowledge, the energy costs of the actin remodeling have not yet been assessed in ectotherms.

The goal of our study was to determine the cellular energy costs of actin remodeling in a common marine bivalve, the Pacific oyster *Crassostrea* [also *Magallana*] *gigas* (Thunberg 1793). Bivalves including oysters are among the few invertebrate ectotherms for which cellular energy budgets have been obtained (Cherkasov et al., 2006; Ivanina and Sokolova, 2008; Pace et al., 2006). Earlier studies focused on the energy costs of protein synthesis, xenobiotic detoxification and compensation of the mitochondrial proton leak in oysters (Cherkasov et al., 2006; Ivanina and Sokolova, 2008; Pace et al., 2006). We used a common approach for assessing the energy requirements of actin maintenance by measuring cellular oxygen consumption in the presence and absence of specific inhibitors of actin remodeling. This approach was successfully applied to mammalian cells (Bernstein and Bamburg, 2003; Engl et al., 2017). We chose three specific inhibitors of actin treadmilling with different mechanisms of action: latrunculin B, jasplakinolide and cytochalasin D (Bernstein and Bamburg, 2003; Brown and Spudich, 1979; Bubb et al., 1994; Wakatsuki et al., 2001). Latrunculin B binds to actin monomers hindering their incorporation into actin polymer, jasplakinolide binds laterally to mature actin filaments and prevents polymer disassembly, whereas cytochalasin D caps the barbed (plus) end of actin filaments and prevents further polymerization (Goddette and Frieden, 1986). Our study indicated low energy costs of actin maintenance under normal physiological conditions in oyster cells but found an unexpected stimulatory effect of cytochalasin D on mitochondrial proton leak. This finding highlights the potential importance of the actin cytoskeleton in regulation of cellular metabolism and warrants further investigation of the mechanisms that link suppression of actin polymerization with increased mitochondrial oxygen demand.

## MATERIALS AND METHODS

### Materials

Latrunculin B was purchased from AdipoGen AG (Liestal, Switzerland), ouabain from Sigma-Aldrich (St Louis, MO, USA), and jasplakinolide and cytochalasin D from Cayman Chemical (Ann Arbor, MI, USA). All other chemicals were purchased from Sigma-Aldrich or Fisher Scientific (Schwerte, Germany) and were of analytical grade or higher.

### Animal collection and maintenance

Adult oysters were collected in the German Wadden sea in low intertidal zone and brought to the University of Rostock, Germany, within 48 h. For acclimation to laboratory condition the animals were kept at least 2 weeks in recirculated temperature-controlled aquarium systems (Kunststoff-Spranger GmbH, Plauen, Germany) with artificial sea water (Tropic Marin^®^, Wartenberg, Germany) adjusted to salinity 33±1 and temperature 15±0.5°C. Animals were fed *ad libitum* by continuous addition of a commercial live algal blend (DTs Premium Blend Live Marine Phytoplankton, Coralsands, Mainz Castel, Germany) according to the manufacturer's instructions.

### Cell isolation

Mantle and gill tissues were excised on ice and subsequently washed in calcium- and magnesium-free saline (CMFS) at an osmolarity of 1100 mOsm. The CMFS buffer contained 20 mmol l^−1^ 4-(2-hydroxyethyl)-1-piperazineethanesulfonic acid (HEPES) at pH 7.5, 500 mmol l^−1^ NaCl, 12.5 mmol l^−1^ KCl and 5 mmol l^−1^ ethylenediaminetetraacetic acid (EDTA). Each tissue was finely chopped using scissors in 10 ml of fresh CMFS buffer, yielding fragments of 1–3 mm in size. The resulting tissue suspension was centrifuged at 1000 ***g*** for 2 min at 4°C, after which the supernatant was discarded, and the pellet was resuspended in 20 ml of fresh CMFS buffer. Cells were dissociated by gentle stirring at room temperature for 1 h. The homogenate was then filtered through a 100 µm strainer and subjected to a second centrifugation at 300 ***g*** for 5 min at 4°C to obtain a cell pellet. The pellet was resuspended in oyster physiological saline (OPS, ∼1050 mOsm) containing 20 mmol l^−1^ HEPES pH 7.5, 500 mmol l^−1^ NaCl, 20 mmol l^−1^ KCl, 1 mmol l^−1^ MgSO_4_, 5 mmol l^−1^ ethylene glycol-bis(2-aminoethylether)-N,N,N′,N′-tetraacetic acid (EGTA), and 5 mmol l^−1^ glucose. Cell viability was routinely assessed by Trypan Blue staining and was >90% in all cell suspensions.

### Mitochondrial isolation

Mitochondria were isolated from oyster gill tissues following the earlier established protocol (Sokolov and Sokolova, 2019). In brief, 1–2 g of gill tissue was homogenized in ice-cold buffer comprising 100 mmol l^−1^ sucrose, 200 mmol l^−1^ KCl, 100 mmol l^−1^ NaCl, 8 mmol l^−1^ EGTA, 50 µg l^−1^ aprotinin, 1 mmol l^−1^ phenylmethylsulfonyl fluoride (PMSF) and 30 mmol l^−1^ HEPES, pH 7.5. Homogenization was performed using several passes of a Potter–Elvehjem homogenizer at 200 r.p.m. The resulting homogenate was centrifuged at 2000 ***g*** for 4 min at 4°C to remove cellular debris. The supernatant was then centrifuged at 8500 ***g*** for 8 min at 4°C to isolate the mitochondrial pellet. The mitochondria were washed once with isolation buffer and collected by a brief centrifugation (8500 ***g*** for 5 min). The final mitochondrial pellet was resuspended in ice-cold assay medium containing 440 mmol l^−1^ sucrose, 130 mmol l^−1^ KCl, 10 mmol l^−1^ NaCl, 30 mmol l^−1^ HEPES pH 7.2, 10 mmol l^−1^ glucose, 1 mmol l^−1^ MgCl_2_, 10 mmol l^−1^ KH_2_PO_4_ and 1% BSA.

### Determination of oxygen consumption rates

Oxygen consumption rates (*Ṁ*_O_2__) of isolated cells and mitochondria were measured at 20°C using a high-resolution Oxygraph 2k respirometer (Oroboros, Innsbruck, Austria) with integrated DatLab 6 software. To assess the energy demands associated with actin maintenance, cellular oxygen consumption was measured both in the presence and absence of specific actin remodeling inhibitors at 15°C. Prior to experimentation, a two-point calibration for 0% and 100% air saturation was conducted. A 200 µl suspension of cells was added to the respirometer chamber containing 1.8 ml of glucose-supplemented OPS at 15°C, and routine oxygen consumption was recorded. Subsequently, the following compounds were sequentially introduced into the chamber, with final concentrations as indicated: ouabain (2.7 mmol l^−1^) to inhibit sodium-potassium ATPase (NKA), cytochalasin D (24.6 µmol l^−1^), jasplakinolide (14 nmol l^−1^) and latrunculin B (2.53 µmol l^−1^) (Bernstein and Bamburg, 2003; Brown and Spudich, 1979; Bubb et al., 1994; Wakatsuki et al., 2001). To inhibit cytochrome c oxidase (CCO) and assess residual non-mitochondrial respiration, 40 mmol l^−1^ sodium azide was added in the final step. The addition of ouabain was used to reduce the background respiration of the cells, allowing for better detection of the subtle contribution of actin treadmilling inhibition to oxygen consumption. The sequential addition of cytochalasin D, jasplakinolide and latrunculin B allowed for testing the effects of each individual inhibitor, ultimately resulting in a pharmacological cocktail that effectively arrests actin dynamics in living cells (Peng et al., 2011). Stock solutions of ouabain and sodium azide were prepared in water. Cytochalasin D was dissolved in DMSO, and jasplakinolide and latrunculin B were dissolved in ethanol. The final concentration of ethanol was <0.1%, which had no significant effect on respiration (data not shown). Sample size was 5 for the gill and the mantle cells, each sample comprising isolated cells from a different oyster.

Since cytochalasin D was the only inhibitor that elicited significant effects on cellular respiration, we conducted a second experiment to compare cellular respiration under different substrate and inhibitor conditions in the presence of cytochalasin D or dimethyl sulfoxide (DMSO) as a vehicle control. Oxygen consumption was measured in parallel using two 2 ml chambers, each containing 200 µl of the same cellular suspension of isolated gill cells at 20°C. Cellular respiration was assessed sequentially under the following conditions: (1) routine respiration with glucose as the sole substrate; (2) respiration with 5 mmol l^−1^ pyruvate (complex I) supplied with 2 mmol l^−1^ malate to initiate respiration; (3) respiration with the addition of 10 mmol l^−1^ succinate to drive electron flow through complex II; (4) measurement of proton leak in the LEAK state following the addition of oligomycin, an F_o_, F_1_-ATPase inhibitor; (5) maximal electron transport system (ETS) capacity, achieved by the addition of 7.5 mmol l^−1^ carbonyl cyanide m-chlorophenyl hydrazone (CCCP) to uncouple the mitochondria; (6) inhibition of complex I by 1 µmol l^−1^ rotenone; (7) inhibition of complex III by 2.5 µmol l^−1^ antimycin A, followed by inhibition of complex IV with 40 mmol l^−1^ sodium azide to quantify non-ETS oxygen consumption (Divakaruni and Jastroch, 2022). Molluscan gill cells possess the ability to uptake dissolved organic matter, including organic acids, through their intact membranes (Baines et al., 2005; Griffin et al., 2023; Sepers, 1977; Sorokin and Wyshkwarzev, 1973). Therefore, there was no need to permeabilize the cell membrane in our experiments to ensure substrate entry. Sample size was 8, each sample comprising isolated cells from a different oyster. Protein concentrations in the cellular suspensions were determined using the Bradford assay (Bio-Rad, Hercules, CA, USA). The average protein concentration in the respirometry chambers was 1.37±0.16 mg ml^−1^. Cellular oxygen consumption rates were normalized to protein content and expressed as nmol O_2_ s^−1^ g^−1^ protein.

Based on the respirometry assays conducted, the following cellular respiration parameters were determined: (1) routine respiration (RR) utilizing glucose; (2) RR with pyruvate supplementation; and (3) RR with combined supplementation of succinate and pyruvate. These parameters reflect the total oxygen consumption of intact cells, encompassing both mitochondrial and non-mitochondrial respiration. Furthermore, the mitochondrial proton leak rate and ETS activity were assessed, corresponding to oligomycin-inhibited respiration and CCCP-uncoupled respiration in intact cells, respectively. To isolate mitochondrial-specific respiration, these measurements were corrected for non-mitochondrial oxygen consumption determined in the presence of antimycin A.

In addition, oxygen consumption in isolated mitochondria was measured using an Oxygraph 2k high-resolution respirometer (Oroboros Instruments, Innsbruck, Austria) at 20°C, following a two-point calibration for 0% and 100% air saturation. Mitochondria were isolated as described elsewhere (Sokolov and Sokolova 2019). Cytochalasin D was added at a final concentration of 24.6 µmol l^−1^, while the control received an equivalent volume of DMSO. The substrate–uncoupler–inhibitor titration (SUIT) protocol used the following sequential additions: (1) 5 mmol l^−1^ pyruvate and 2 mmol l^−1^ malate to stimulate complex I respiration; (2) 10 mmol l^−1^ succinate to promote electron flow through complex II; (3) 2.5 mmol l^−1^ ADP to achieve state 3 respiration (OXPHOS state); (4) 2.5 µmol l^−1^ oligomycin to induce state 4o respiration (LEAK state); (5) 7.5 mmol l^−1^ CCCP to measure the uncoupled ETS activity; (6) 1 µmol l^−1^ rotenone to inhibit complex I activity; (7) 2.5 µmol l^−1^ antimycin to inhibit complex III activity; (8) 5 mmol l^−1^ N,N,N′,N′-tetramethyl-p-phenylenediamine (TMPD) and 2 mmol l^−1^ ascorbate to assess complex IV (cytochrome *c* oxidase, CCO) activity, followed by inhibition of CCO using 40 mmol l^−1^ azide. Sample size was 5, each sample comprising isolated mitochondria from the gills of a different oyster.

Mitochondrial respiratory states and control indices were determined following established methodologies (Estabrook, 1967; Gnaiger, 2020). Oxidative phosphorylation (OXPHOS) flux was calculated based on the ADP-stimulated respiration rate, indicative of ATP synthesis capacity. Respiration in the LEAK state, determined in the presence of oligomycin, served as a measure of the mitochondrial proton leak rate, reflecting the ETS activity necessary to counterbalance futile proton and cation cycling when ATP synthesis is inhibited (Rolfe and Brand, 1997). LEAK respiration was analyzed using substrates specific to complex I (pyruvate, LEAK I) as well as a combination of complex I and complex II substrates (pyruvate and succinate, LEAK I+II). ETS capacity was measured as CCCP-uncoupled respiration and evaluated using substrates targeting complex I (pyruvate, ETS I), complex II (succinate, ETS II), and a combination of both (ETS I+II).

Protein concentrations in the cellular suspensions were determined using the Bradford assay (Bio-Rad, Hercules, CA, USA). The average protein concentration in the respirometry chambers was 1.37±0.16 mg ml^−1^. Oxygen consumption rates of isolated mitochondria exposed to cytochalasin D were expressed as a percentage of the vehicle control.

### Immunoblotting

To assess whether the effects of cytochalasin D on cellular respiration are mediated by changes in actin associated with mitochondria, we quantified mitochondria-bound actin in control and cytochalasin D-treated cells using immunoblotting. While this method does not measure actin biosynthesis or degradation rates, it provides a snapshot of mitochondria-bound actin after treatment. Oyster gills (approximately 1 g) were excised, placed in CMFS buffer (1100 mOsm) and finely chopped. The resulting mixture was washed once with the same buffer and incubated for 1 h in a fresh aliquot of CMFS buffer supplemented with 0.1% trypsin and 1 mg ml^−1^ collagenase (final concentrations) at room temperature (RT). Following digestion, the tissue was filtered through a 100 μm cell strainer, and the cell suspension was collected by centrifugation at 2000 ***g*** for 5 min at 4°C. The resulting cell pellet was resuspended in 4 ml of CMFS, and divided into two fractions: control and experimental. An aliquot of cells was stained with Trypan Blue to assess viability, which exceeded 90% in all isolations (data not shown). The experimental fraction was treated with cytochalasin D to a final concentration of 25 μM, and both samples were incubated on ice for 2 h. The cell suspensions were diluted to 10 ml with ice-cold CMFS and subjected to nitrogen cavitation at 500 psi for 5 min using a Cell Disruption Vessel (Model 4639, Parr Instrument Company, Moline, IL, USA). The resulting suspension was centrifuged for 5 min at 2400 ***g*** to precipitate cellular debris. The supernatant was then recentrifuged at 8500 ***g***, and the mitochondrial pellet was collected in 1% sodium dodecyl sulfate (SDS) as a lysis buffer. Lysis was conducted on ice for 2 h. Proteins were resolved by SDS-PAGE, transferred to a polyvinylidene difluoride (PVDF) membrane (Merck KGaA, Darmstadt, Germany) and probed with either anti-aconitase antibody (Cell Signaling, cat. no. 6571) or anti-actin antibody (Sigma-Aldrich, Merck KGaA, Darmstadt, Germany, cat. no. A2066). Although the anti-actin antibody recognizes both G- and F-actin, the mitochondrial isolation procedure eliminates all cytosolic components, including non-polymerized G-monomers. As a result, only F-actin bound to mitochondria is retained in the mitochondrial preparation. Signals were detected using X-ray film, which was subsequently scanned using a transmission scanner. The resulting bands were quantified using GelQuant.NET software (biochemlabsolutions.com). Sample size was 6, each sample comprising isolated mitochondria from the gills of a different oyster.

### Calculations and statistics

The E-L coupling efficiency (E-L CE) was calculated as 1–(LEAK/ETS), where LEAK represents the respiration rate attributable to proton leak, and ETS is the uncoupled electron transport system activity (Gnaiger, 2020). The E-L CE ranges from 0.0, indicating zero coupling (LEAK=ETS), to 1.0, reflecting a fully coupled system (LEAK=0). The data were tested for normality using Kolmogorov–Smirnov test. Oxygen consumption rates and respiratory ratios, including E-L CE, the ratio of proton leak (LEAK) to routine respiration (routine metabolic rate, RMR), the ratios of LEAK:ETS and RMR:ETS, and the ratio of actin to aconitase protein levels (based on immunoblotting) were compared between cells treated with inhibitors or vehicle control using two-tailed ratio paired *t*-tests. The *t*-tests evaluated deviations in mitochondrial respiration, expressed as a percentage of the vehicle control (DMSO), from a fixed reference value of 100%. Statistical analyses were performed using GraphPad Prism v. 7.05 (GraphPad Software, La Jolla, CA, USA). For all comparisons, a significance level of *P*<0.05 was used to determine whether the treatments induced statistically significant changes in respiration.

## RESULTS

### Isolated cells

**Fig. 1.**
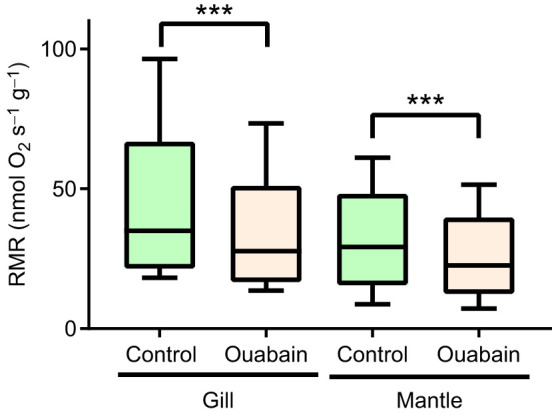
**Effect of the NKA inhibitor ouabain on the routine metabolic rate (RMR) of isolated gill and mantle cells of *Crassostrea*** ***gigas*****.** Asterisks indicate significant differences (***P<0.001). *N*=5. Boxes in all figures show the 25–75th percentiles with median; whiskers show the 1.5× interquartile range.

Addition of ouabain led to a significant decrease in the routine respiration rate of isolated oyster cells (Fig. 1). Generally, oxygen consumption related to the NKA activity accounted for 23% and 19% of the routine cellular respiration in the gill and the mantle cells, respectively. Addition of the actin polymerization inhibitors, jasplakinolide and latrunculin B had no significant effect on cellular respiration (*P*>0.05; data not shown). The upper limit of estimated energy cost of actin polymerization assessed by a decrease in the respiration rate upon both jasplakinolide and latrunculin B addition was ∼1.3% and 2.5% in the gill and the mantle cells of *C. gigas*, respectively.

**Fig. 2.**
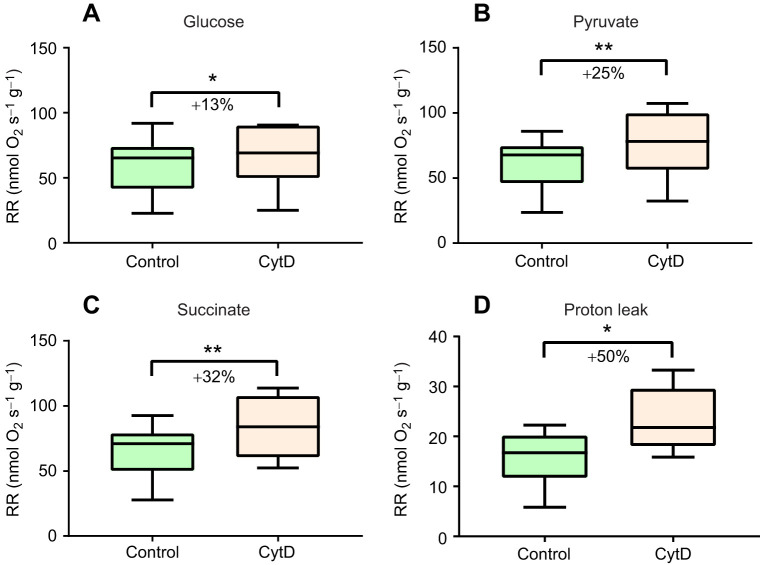
**Effect of cytochalasin D (CytD) on the oxygen consumption rates of isolated gill cells of *C. gigas* with different substrates and with oligomycin.** (A) Routine respiration rate (RR) with glucose. (B) RR with pyruvate supplementation. (C) RR with combined succinate and pyruvate supplementation. (D) Proton leak rate with the F_o_, F_1_-ATPase inhibitor, oligomycin. Asterisks indicate significant effects of CytD (*P<0.05, **P<0.01). *N*=8.

Unexpectedly, addition of cytochalasin D led to a significant increase in the RMR of the oyster gill and mantle cells (Fig. 2, data shown for the gill cells only). In the gill cells, the routine cellular respiration with endogenous substrates was stimulated by ∼13% by cytochalasin D (Fig. 2A). In the presence of exogenous substrates, pyruvate and succinate, the addition of cytochalasin D increased cellular respiration by 25–32% (Fig. 2B,C). Furthermore, cytochalasin D significantly increased mitochondrial proton leak in oyster gill cells by 50% (Fig. 2D). This enhancement led to a rise in the contribution of proton leak to routine cellular respiration, increasing from 31% in control cells to 50% in cytochalasin D-treated cells. Non-mitochondrial respiration accounted for an average of 13.5% of total cellular oxygen consumption and was not significantly altered by treatment with cytochalasin D (*P*>0.05).

**Fig. 3.**
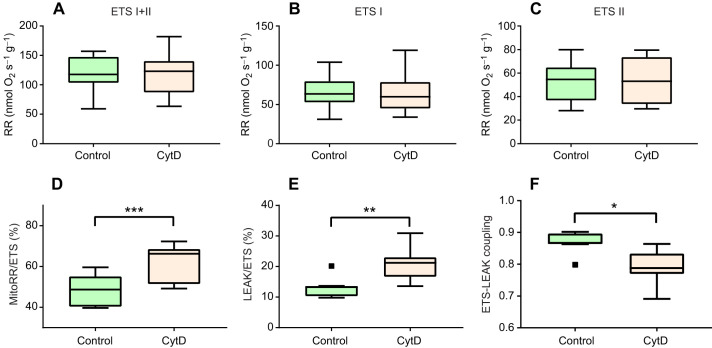
**Effect of CytD on the uncoupled respiration rate, ratio of routine mitochondrial respiration, proton leak to ETS activity and ETS-LEAK coupling ratio of isolated gill cells of *C. gigas*.** (A) Electron transport system (ETS) activity with complex I and II substrates. (B) ETS activity with complex I substrate. (C) ETS activity with complex II substrate. (D) Ratio of mitochondrial respiration rate to uncoupled ETS activity (%). (E) Proton leak to ETS ratio (%). (F) ETS-LEAK coupling. Asterisks indicate significant effects of CytD (*P<0.05, **P<0.01, ***P<0.001). *N*=8.

However, cytochalasin D had no effect on the mitochondrial ETS activity in the isolated gill cells (Fig. 3A–C). This held true for the total ETS activity and the ETS flux through complexes I and II, assessed separately. Exposure to cytochalasin D increased the ratio of the routine mitochondrial respiration to the total ETS capacity from ∼48 to 62% (Fig. 3D), and the ratio of the proton leak to the total ETS capacity from ∼13 to 21% (Fig. 3E). As a result, the E-L coupling efficiency decreased from 0.87 to 0.79 in the presence of cytochalasin D (Fig. 3F).

### Isolated mitochondria

**Fig. 4.**
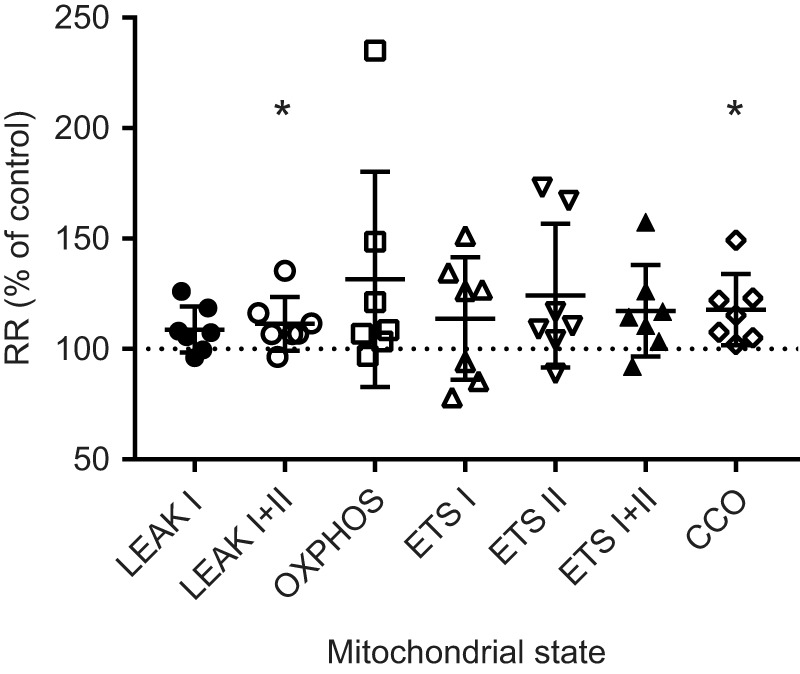
**Effect of CytD on the respiration rate of isolated gill mitochondria of *C. gigas*.** The *x*-axis shows different mitochondrial states: LEAK I (proton leak rate with pyruvate), LEAK I+II (proton leak rate with pyruvate and succinate), OXPHOS (ADP-stimulated respiration with pyruvate and succinate), ETS I+II (uncoupled ETS activity with pyruvate and succinate), ETS I (uncoupled ETS activity with pyruvate alone), ETS II (uncoupled ETS activity with succinate alone) and CCO (cytochrome *c* oxidase activity). Respiration rates of isolated mitochondria under each state, measured in the presence of CytD, are expressed as percentages relative to the vehicle control for the same mitochondrial isolate. Asterisks indicate significant effects of CytD (*P<0.05). Error bars indicate means±s.e.m.; *N*=5.

Addition of cytochalasin D had no effect on the baseline respiration of isolated gill mitochondria from oysters respiring on NADH-linked substrates (pyruvate and malate) (*P*>0.05, LEAK I, Fig. 4). Mitochondrial baseline respiration with NADH- and FADH_2_-linked substrates increased by 11% following the addition of cytochalasin D (*P*<0.05, LEAK I+II, Fig. 4). In contrast, OXPHOS respiration and ETS activity, whether assessed globally or individually using NADH-linked (ETS I) and FADH_2_-linked (ETS II) substrates, did not exhibit significant changes in response to cytochalasin D (*P*>0.05). However, CCO activity increased by 18% compared with the vehicle control (*P*<0.05, Fig. 4).

**Fig. 5.**
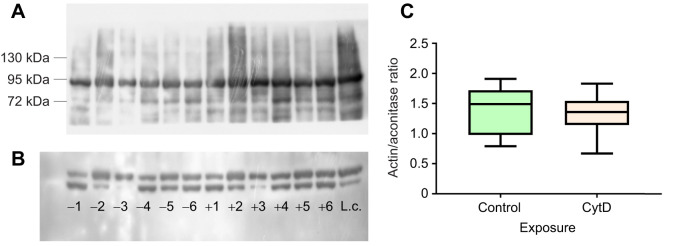
**Immunoblot analysis and quantification of the actin-to-aconitase ratio in mitochondria isolated from *C. gigas* gill cells incubated with either DMSO (vehicle control) or cytochalasin D.** (A,B) Immunoblot analysis of protein levels of aconitase (A) and actin (B). Negative sample numbers represent the vehicle control, while positive numbers correspond to cytochalasin D-treated cells from the same isolate. L.c., loading control. (C) Quantification of the actin-to-aconitase ratio in mitochondria isolated from *C. gigas* gill cells incubated with either DMSO (vehicle control) or cytochalasin D. No significant differences in the actin-to-aconitase ratio were observed between control and cytochalasin D-treated cells (ratio paired *t*-test, *P*>0.05). *N*=6.

Immunoblotting analysis revealed no significant effect of cytochalasin D incubation on the amount of actin associated with mitochondria in gill cells (Fig. 5). It is important to note that this analysis only accounts for actin that is tightly associated with mitochondria and therefore retained during the mitochondrial isolation process.

## DISCUSSION

Our study using specific inhibitors of actin remodeling did not yield evidence supporting substantial energetic costs associated with the maintenance of the actin cytoskeleton in mantle and gill cells of the Pacific oyster, *C. gigas*, under control conditions. The estimated energy consumption attributed to actin remodeling processes disrupted by the addition of jasplakinolide and latrunculin B was approximately 1.3% in gill cells and 2.5% in mantle cells of *C. gigas*. These low estimates stand in stark contrast to those reported for mammalian and avian neurons, where actin remodeling accounts for 25–50% of cellular oxygen consumption (Bernstein and Bamburg, 2003; Engl et al., 2017), as well as for platelets and immune cells, where it has been estimated to represent 50 and 73% of cellular energy demand (Daniel et al., 1986; Roos et al., 1976). In plants and yeast, ATP deficiency has been demonstrated to significantly slow down actin remodeling, resulting in considerable energy savings (Dai et al., 2022). The discrepancy between these findings and our estimates suggests that, in the gill and mantle cells of adult mollusks such as *C. gigas*, cytoskeletal dynamics – particularly actin remodeling – are not highly energy-intensive processes under normal physiological conditions. To the best of our knowledge, these are the only published estimates of the energy costs associated with actin remodeling in invertebrates or ectotherms, limiting the ability to draw broader generalizations. The substantial differences in estimated energy costs between oyster cells and those reported for mammalian and avian cells using similar methodologies (Bernstein and Bamburg, 2003; Daniel et al., 1986; Engl et al., 2017; Roos et al., 1976) are intriguing and warrant further investigation to determine whether they are tissue- and species-specific or reflect broader differences between endotherms and ectotherms. A key limitation of this and similar studies is that the energy cost estimates consider only the ATP demand for actin treadmilling, excluding the likely significant costs of actin biosynthesis and degradation, which may increase the total energy expenditure for actin cytoskeleton maintenance.

Actin plays a crucial role in regulating the mitochondrial network, influencing processes such as mitochondrial fission, mitophagy, metabolism and membrane potential (Fung et al., 2023). Actin polymerization has been shown to stimulate Ca²^+^ entry into mitochondria, with this Ca²^+^ influx being inhibited by latrunculin A (Chakrabarti et al., 2018). Mitochondrial Ca²^+^ uptake, in turn, stimulates ETS activity and enhances mitochondrial membrane potential (Matuz-Mares et al., 2022). Additionally, actin polymerization around mitochondria helps stabilize mitochondrial membrane potential (Chakrabarti et al., 2018; Fung et al., 2023, 2019). Given this context, the stimulatory effect of cytochalasin D, an inhibitor of actin polymerization, on cellular respiration in oyster gill cells – ranging from 13% to 32% depending on the oxidized substrate – was unexpected. This effect cannot be attributed to direct modulation of mitochondrial ETS activity, as cytochalasin D had no impact on ETS function in isolated mitochondria or intact cells. Instead, the observed increase in respiration appears to be driven by a significant (50%) elevation in mitochondrial proton leak, induced by the disruption of actin cytoskeleton remodeling. This increase in proton leak was also reflected in the enhanced contribution of proton leak to RR in gill cells. Notably, a modest (11%) increase in proton leak was also observed in isolated oyster mitochondria oxidizing both complex I and complex II substrates.

Oxygen consumption in the LEAK state reflects the combined contributions of futile proton and cation cycles, which dissipate the mitochondrial membrane potential without producing ATP (Jastroch et al., 2010). Mitochondrial proton leak consists of two key components: a baseline leak, largely driven by cation co-transport through substrate transporters and the adenine nucleotide translocator (ANT), and an inducible leak, modulated by uncoupling proteins (UCPs). The latter plays a crucial role in regulating mitochondrial reactive oxygen species (ROS) production and, in endothermic organisms, contributes to metabolic heat generation (Hirschenson et al., 2022). Given that proton leak contributes approximately 30% to routine cellular respiration in oyster cells (this study) and ranges from 10 to 40% across various animal cells (Hulbert et al., 2002; Jastroch et al., 2010; Sokolova, 2021), the enhancement of proton leak could have significant implications for the metabolic costs associated with mitochondrial maintenance. The precise mechanisms underlying the cytochalasin D-induced increase in proton leak remain unclear. One potential explanation is that the effects of cytochalasin D are not directly attributable to its impact on the actin cytoskeleton but may instead result from non-specific side effects, such as impaired membrane integrity or altered activity of proteins involved in proton import, including uncoupling proteins. Although cytochalasin D does not share chemical similarities with known mitochondrial uncouplers, this possibility cannot be excluded. This hypothesis may account for the observation that two other disruptors of actin treadmilling, jasplakinolide and latrunculin B, had no effect on mitochondrial proton leakage in oyster cells.

Alternatively, the differential effects of cytochalasin D, jasplakinolide and latrunculin B may be attributed to distinct mechanisms by which these compounds disrupt actin treadmilling (Rotsch and Radmacher, 2000; Wang et al., 2019; White et al., 2001). While both cytochalasins and latrunculins promote actin disassembly, cytochalasin D does so by capping actin filaments while latrunculin sequesters actin monomers (Fujimoto et al., 2000). In contrast, jasplakinolide promotes polymerization and subsequently stabilizes the formed microfilaments, preventing their depolymerization (Trendowski et al., 2014). Previous studies have demonstrated that inhibitors of actin treadmilling, including cytochalasins, jasplakinolide and latrunculins, have different effects on actin-dependent processes such as cell movement, endocytosis, intracellular transport and apoptosis (Fujimoto et al., 2000; Hao et al., 2006; Rotsch and Radmacher, 2000; Trendowski et al., 2014; White et al., 2001). This suggests that the specific mechanism by which the actin cytoskeleton is disrupted, rather than the mere occurrence of its disruption, can have significant consequences for actin-dependent processes, including mitochondria–actin interactions. Further research is needed to assess the generalizability of these findings and to explore in greater depth the potential mechanistic link between various aspects of actin structure and dynamics and mitochondrial proton leak.

Studies in mice show that the inhibition of actin polymerization by cytochalasin B resulted in an elevated oxygen consumption rate in brain mitochondria, which was attributed to increased cytochrome *c* oxidase (CCO) activity (Takahashi et al., 2018). This enhancement in CCO activity is presumed to result from accelerated association and dissociation of the cytochrome *c* oxidase complex with CCO, thereby facilitating CCO function (Takahashi et al., 2018). Similarly, in our study, we observed a modest but statistically significant 18% increase in CCO activity in oyster gill mitochondria following cytochalasin D treatment, suggesting that a comparable mechanism may be involved. In oyster mitochondria, the enhanced CCO activity did not translate into an overall increase in ETS flux, probably because CCO activity is not rate limiting under normal conditions (Adzigbli et al., 2024, 2022; Sokolov and Sokolova, 2019). Interestingly, in rodent and human leukocytes, inhibition of actin remodeling also stimulated oxygen consumption; however, this effect was mediated by an increase in plasma membrane NADPH oxidase activity rather than alterations in mitochondrial activity (Zabucchi et al., 1981). These findings underscore the broader metabolic implications of actin polymerization state (DeWane et al., 2021; Fung et al., 2023), suggesting that studies using actin inhibitors have limitations when assessing the energetic costs of cytoskeleton maintenance in animal cells.
